# Graves’ Disease in a Young Patient With Turner’s Syndrome: The Genetic Association

**DOI:** 10.7759/cureus.35593

**Published:** 2023-02-28

**Authors:** Anirban Majumder, Wesley H Brooks

**Affiliations:** 1 Endocrinology, Kali Prasad Chowdhury Medical College & Hospital, Kolkata, IND; 2 Department of Chemistry, University of South Florida, Tampa, USA

**Keywords:** female, autoimmune disease, x chromosome inactivation, graves’ disease, turner’s syndrome

## Abstract

Introduction: Autoimmune diseases occur more often in females, suggesting a key role for the X chromosome. Curiously, individuals with Turner syndrome (TS), with fewer copies of X-linked genes, are prone to develop autoimmune conditions. Hashimoto's thyroiditis (HT) is described with a relatively high frequency in patients with TS while the association with Graves' disease (GD) is rare. Here we report a rare case of TS with GD in a young patient.

Method: A 14-year-old girl presented with hyperthyroid symptoms and eye signs that developed over the past six months. She had somatic stigmata of TS. TS was diagnosed by karyotyping (45,XO/46,XX del Xq22) and GD was diagnosed by a thyroid function test and the presence of autoantibodies. She was treated effectively with carbimazole for GD. Estrogen replacement therapy was also initiated to induce the development of secondary sex characteristics.

Conclusion: X chromosome inactivation, an epigenetic process that establishes and maintains dosage compensation of X-linked genes, is especially vulnerable to disruption and may contribute to an autoimmune disease process. The occurrence of autoimmune diseases in patients with TS is discussed with regard to possible abnormalities in X-linked dosage compensation.

## Introduction

Turner’s syndrome (TS) is a well-known clinical presentation in females. The karyotype in TS is typically thought of as 45,XO (a.k.a. “classic” TS), but there can be variations, such as mosaics of 45,XO/46,XX, or abnormalities in the second X chromosome, such as 46,XXq- (i.e. deletion of the long arm, Xq, of the second X chromosome). Autoimmunity can be a prominent feature in TS with an increased frequency of autoimmune thyroid disorders (AITD). Celiac disease, vitiligo, psoriasis, type 1 diabetes, adrenocortical insufficiency, juvenile idiopathic arthritis, and inﬂammatory bowel disease are other autoimmune diseases described in patients with TS [1]. Among the AITD, Hashimoto’s thyroiditis (HT) is by far the most common diagnosis. Graves’ disease (GD), another common AITD, is rare in TS. Here, we report a 14-year-old girl with TS who presented with GD. The etiopathogenetic and genetic relationships between these two disorders are difficult to ascertain. TS may be associated with factors that render them susceptible to autoimmune disorders in later life. Some possible explanations are offered to clarify the association.

## Case presentation

A 14-year-old girl presented with arrested growth over the last four years and exophthalmos for six months. On further inquiry, she was found to have primary amenorrhea, loss of weight (despite an increased appetite), frequent bowel movements, palpitation, sweating (especially night sweats), heat intolerance, and proximal muscle weakness (lower limbs > upper limbs). There was no history of diplopia, lowered visual acuity, or disturbed sleep. The girl was the first child of healthy unrelated parents of normal stature, father 165 cm, and mother 152 cm. She was a premature baby (32 weeks) of a teenage mother but no other birth history was available. She had a history of delayed developmental milestones.

On examination, she was found to be 120 cm tall (-5.2 SD), 21 kg in weight (-2.6 SD), with an arm span of 112 cm, but the upper segment was longer (62 cm) than the lower segment (58 cm) and upper to lower segment ratio is 1.07. She had a 112 per min regular pulse and was normotensive. The other features noted were low set ears, micrognathia, shield chest, Tanner’s stage I breast, widely spaced nipples, cubitus valgus, infantile external genitalia, absent axillary and pubic hair, genu valgum, short fourth metatarsals, multiple pigmented naevi. There was no webbing of the neck. Exophthalmos with lid lag and lid retraction was seen, but external ophthalmoplegia and dermopathy were absent. The thyroid was soft, diffusely enlarged (Grade 2), with positive bruit and no retrosternal extension. She was emotionally labile, and restless had proximal muscle weakness and exaggerated deep tendon reflexes. Laboratory investigations of the case are presented in Table 1.

**Table 1:** Laboratory investigations of the case report

Investigation	Results	Normal range
Hemoglobin (Hb)	11.7 gm/dl	12.1 to 15.1 gm/dl
Leucocyte count	6000/mm3	4,500 to 11,000/mm3
Differential count	Normal	
Fasting plasma glucose	75 mg/dl	70- 99 mg/dl
Urea	17 mg/dl	7-21 mg/dl
Creatinine	0.6 mg/dl	0.7-1.3 mg/dl
Follicle-stimulating hormone (FSH)	29.5 IU/L	1-11 IU/L
Luteinizing hormone (LH)	24.1 IU/L	1.9-12.5 IU/L
Prolactin	25.4 ng/ml	3.4-20.3 ng/ml
Estradiol	18 pg/ml	21-160 pg/ml
Karyotype (with 20 metaphase cells analyzed)	Initial: 45,XO; 2nd karyotyping: 45,XO (11)/46,XX del Xq22 (9)	
Thyroid stimulating hormone (TSH)	0.01 uU/ml	0.50-5.50 uU/ml
Free Thyroxine (FT4)	4.20 ng/dl	0.85-1.80 ng/dl
Free Triiodothyronine (FT3)	10.26 pg/ml	2.50-5.50 pg/ml
Technetium 99m scan (Tc 99m)	Diffusely enlarged thyroid with uniformly increased uptake of 2.42%	0.5-1.4%
Anti-TSH receptor antibody (TRAb)	3.12 iu/L (Positive)	1.22-1.58 iu/L
Anti-thyroperoxidase (anti-TPO) antibody	375 iu/ml (Positive)	normal 0-35 iu/ml
Antithyroglobulin antibody (anti-TG)	42 iu/ml (Negative)	0-125 iu/ml
Electrocardiography (ECG)	Sinus tachycardia	-
Ultrasonography of pelvis	Rudimentary uterus; ovaries not visualized	
Computed tomography (CT) of orbits	No retro-orbital mass, no enlargement of extraocular muscle bellies and mild increase in retro-ocular orbital fat bilaterally	-

The laboratory findings were compatible with the diagnosis of GD. Treatment with 20 mg carbimazole was started. She responded well with normalization of pulse rate, improvement in eye signs, 0.5 kg gain in weight, and improvement in proximal muscle weakness. Her thyroid function normalized within four months when the dose gradually decreased to 5 mg daily maintenance dose in the next two months and as expected, no change in her secondary sex characteristics was observed. Thyroid stimulating hormone (TSH)-receptor antibody (TRAb) was not measured at the end of the therapy which often relates to the risk of recurrence. She remained euthyroid with a 5 mg maintenance dose in the next two years follow-up. She was given oral ethinyl estradiol, starting with a 5 ug dose and gradually up-titrating to 50 ug daily. Transdermal estrogen is not available and oral ethinyl estradiol was chosen instead. At the latest follow-up (16 years of age), the patient’s breasts had developed to Tanner stage 3. At this point, we have planned to add progesterone therapy. 

## Discussion

Women with TS have 45,X karyotype with no mosaicism (almost 40%) or long arm isochromosome (almost 10%), or mosaicism (45,X/46,XX) and a variety of X structural defects (deletions, ring chromosome, etc) in another 50% [1]. This 14-year-old girl of TS had short stature, primary amenorrhea, and many other somatic stigmata. Her high gonadotropin levels, rudimentary uterus, and 45,XO/46,XX del Xq22 karyotype confirmed the diagnosis of non-classic TS. She developed GD during the last six months for which she sought treatment at our institute. Her clinical presentation especially exophthalmos, thyrotoxic range of thyroid hormones level, uniformly increased uptake on Technetium-99m thyroid scan and high titers of anti-TSH receptor antibody and anti-thyroid peroxidase (TPO) antibody established the diagnosis. The risk of autoimmune diseases in patients with TS is approximately twice compared to the general female population and the excess of autoimmune antibodies (anti-TSH receptor antibody and anti-TPO antibody) is likely to result from the X chromosome defects [1].

Most GD cases are reported in adults and are uncommon in children. A 14-year-old girl with GD is uncommon in our clinical practice. Regardless of the rarity of presentation, proper early diagnosis of the disease is of utmost importance for correct management. The optimal treatment for Graves' disease in children remains under debate. Antithyroid drugs (ATD), thyroid surgery, and radioactive iodine are treatment options in adults [2]. ATD therapy is commonly recommended as the initial treatment for children and adolescents. This girl was treated with carbimazole and to which she responded. Methimazole is the active metabolite of carbimazole and because of its long half-life, is effective as a single daily dose and is particularly helpful in children. Medical therapy with thionamides has potentially serious complications such as agranulocytosis, neutropenia, and drug-induced hepatitis [2]. ATD-induced agranulocytosis develops because of direct toxicity or immune-mediated toxicity and that of severe side effects is very low with the usual methimazole dose of 15 mg/day or less [3]. The thyroid hormone levels usually normalize in four weeks as was found in our patient. She also received propranolol (β-blocker) twice daily for her symptoms during the first four weeks of management and then stopped when she became euthyroid. She experienced no relapse in the next year despite the treatment being tapered and then stopped in six months. Prepubertal patients tend to have a severe course and are more likely to experience medical treatment failure [2]. Some children require more prolonged use of ATD (at least two to four years) to achieve remission. Compliance is therefore an important issue in management [2]. TS is associated with an increased frequency of many autoimmune disorders, namely AITD, celiac disease, inﬂammatory bowel disease, vitiligo, psoriasis, type 1 diabetes, adrenocortical insufﬁciency, juvenile idiopathic arthritis, and rheumatoid arthritis [1]. HT is the leading (about 37%) autoimmune disorder among AITD [1]. GD, another AITD, has been reported only rarely. The underlying immunopathogenic mechanism for this difference remains unexplained but, as HT is one of the more commonly encountered thyroid disorders seen in the general population, it may be so in TS as well. However, the relative risk for GD in TS is about 2.1-fold higher than the general female population and seven times higher for HT than the general female population [1].

Although the pathogenetic mechanisms of autoimmune diseases have become better understood, the reasons for the female preponderance of these conditions remain unclear. The most intriguing theories to explain this are related to ovarian hormones, pregnancy, skewed X chromosome inactivation patterns, defects in the sex chromosomes, and X chromosome gene dosage changes [4]. But most of the autoimmune conditions in TS occur before estradiol exposure [5]. With the current knowledge, it is difficult to explain the higher prevalence of autoimmune diseases in TS where the ovarian hormone level is low and the X chromosome gene dosage is also suspected to be low. The association between certain karyotypes in TS and autoimmune disease has yielded conflicting results. Though an increased risk of autoimmune thyroid disease among women with the isochromosome Xq karyotype has been demonstrated but this notion of a specific association was not observed in the successive studies [1].

The prevalence of HT is also increased in primary ovarian insufficiency (POI) patients with lower estrogen exposure relative to the general population of women. So estrogen exposure or pregnancy cannot explain the greater risk for autoimmunity in TS or primary ovarian insufficiency [6]. On the other hand, androgen deficit, present in both HT and primary ovarian insufficiency, may be proposed as a potential explanation, consistent with the protective role of androgen for suppressing autoimmunity in experimental systems [6]. But women with TS have a much higher prevalence of a variety of autoimmune conditions than those with primary ovarian insufficiency and androgen deficiency alone cannot explain this. Therefore, X chromosome-related gene(s) are expected to play a prominent role in the pathogenesis of autoimmune conditions in TS. The association of the X-isochromosome karyotype among women with TS with excess autoimmunity suggests that a gene on the long arm of the X chromosome (Xq) may play an important role in the development of the autoimmune disease [7]. But our patient had no X-isochromosome karyotype and other studies have failed to confirm this association [5]. Evidence shows that the prevalence of autoimmune disease in the general population of women is 5.8%, for women with POI 15%, and for women with TS 37% [6]. X chromosome abnormalities in the form of microdeletions, permutations, and mutations of genes are established factors in the origin of POI [8,9]. A stepwise decrease in the dosage of X chromosome genes is observed from the general population of women (two normal X chromosomes) to women with POI (macro-microdeletion of the X chromosome) to women with TS (complete absence of one X chromosome or a portion of the X chromosome) [6]. This indicates the relationship between the dosage of X-chromosome genes and autoimmune diseases.

In contrast, female preponderance in autoimmune diseases is evident by the fact that almost 80% of autoimmune patients are female. Klinefelter syndrome (47,XXY), with an extra X chromosome, is found in excess among men with systemic lupus erythematosus (SLE), another autoimmune disease, and the calculated prevalence of SLE among Klinefelter syndrome patients is similar to the prevalence in 46,XX women [10]. The presence of a third X chromosome (Trisomy X, 47,XXX) is not associated with any recognizable abnormalities in sex hormone levels, sexual development, fertility, or pregnancy. But the estimated prevalence of SLE and Sjögren's syndrome (SjS) in women with 47,XXX is 2.5 to 3 times higher than in 46,XX women, and the X chromosome dosage contributing to the prevalence of SLE is independent of hormonal effects [11].

The X chromosome contains around 1,100 genes and spans 155 million deoxyribonucleic acids (DNA) base pairs. Most of the genes on the X chromosome are not sex-specific and, therefore, females only need one active X chromosome. Early in embryonic development, one of the two X chromosomes in females is randomly chosen to remain active and any other X chromosomes are inactivated to ensure dosage compensation for comparable X-linked gene expression between XX females and XY males. This phenomenon is called X-chromosome inactivation (XCI) or lyonization (named after geneticist Mary Lyon) [12]. But 15-25% of the X-linked genes are not silenced or later escape silencing leading to higher expression of those X-linked genes and this can affect the immune response and contribute to autoimmune diseases in females [13]. These data suggest that the dosage of X chromosome genes is a major determining factor for autoimmune diseases. Therefore, a stepwise increase in autoimmune diseases is observed relative to a progressive increase in the gene dosage of the X chromosome (from the general population of women to women who have some genes that escape the customary X-chromosome inactivation, to women with 47,XXX) [4]. The X chromosome, compared to other chromosomes, contains the largest number of genes involved in immune responses which adds to the suspicion of X chromosome involvement in autoimmune diseases [13]. For example, the gene CD40LG, located on the X long arm at Xq24, is overexpressed in systemic sclerosis (SSc) [14]. CD40LG codes for CD40L, a surface protein involved in the interaction of T cells with antigen-presenting cells. Epigenetic dysregulation leading to demethylation of the CD40LG gene is suspected of causing oversensitive T cells in SLE [15]. Also on the X chromosome, TLR7 and TLR8, which are associated with both innate and adaptive immune responses, are located on the short arm of the X chromosome at Xp22 [16]. The copy number, which can influence the dosage, of TLR7 and CD40L genes can impact the immune response [17].

This patient presents a very interesting scenario with regard to X-linked genes, particularly in the 46,XX del Xq22 cells. The deletion from Xq22 is in a common fragile site, FRAXC. Fragile sites can be thousands to hundreds of thousands of base pairs. They contain DNA sequences that can more readily form strand separation which can lead to single- and double-strand breaks, deletions, mutations, insertions, and even viral insertion. And sequences in fragile sites can more readily form problematic alternate DNA conformations and ribonucleic acid/deoxyribonucleic acid (RNA/DNA) hybrids referred to as R loops [18]. The cause of the extensive deletion in this patient is not known, but some of the consequences can be predicted. The X inactivation center at Xq13 may still be intact in both X chromosomes in the 46,XX del Xq22 cells. Therefore, XCI, a random choice as to which X chromosome will be inactivated, may potentially still occur in these cells. However, the missing portion of the X chromosome involves many important genes. Three genes that have been associated with autoimmune diseases are the aforementioned CD40LG, interleukin-1 receptor-associated kinase 1 (IRAK1) at Xq28; and methyl capping protein 2 (MeCP2) also at Xq28 [19]. IRAK1 is a kinase that associates with the interleukin-1 receptor and is involved in the upregulation of NF-kappa B in T cells. Therefore, IRAK1 is important in T cell activation in autoimmune disease. MeCP2 provides another level of epigenetic control by blocking methylated DNA from further modification, such as demethylation or hydroxymethylation. Loss of MeCP2 expression could allow abnormal reactivation of genes. This could include partial or complete loss of XCI with overexpression of X-linked genes.

XCI in female cells is a pivotal epigenetic mechanism [20]. Disruption or failure of this important epigenetic event can result in improper X-linked gene expression and product levels. Normally in the X inactivation process, which occurs early in embryogenesis, the two X chromosomes colocalize in female cells so that their X inactivation centers at Xq13 are nearby. Both X inactivation centers are expressing two non-coding, nuclear-localized RNAs: the X inactivation specific transcript (XIST) and the anti-sense to XIST RNA (TSIX) transcript. XIST and TSIX counteract each other by hybridization and subsequent degradation. But, at some point through a stoichiometric process involving short sense and anti-sense RNAs in the XCIs, the future inactive X (Xi) shuts off TSIX and persists in expressing XIST. Eventually, the active X chromosome (Xa) shuts off both XIST and TSIX expression. XIST remains in the nucleus, binds contiguous chromatin (i.e. the Xi) primarily at LINE-1 elements along with the Xi, and recruits chromatin silencing factors (e.g. DNA methyltransferases, histone deacetylases) [21]. Failure to establish XCI or escape from XCI can lead to higher expression of X-linked genes and may have a role in the higher occurrence of autoimmune diseases in females as seen in SLE and Sjogren's syndrome [11].

Coming back to the current case of non-classic TS with a 45,XO/46,XX del Xq22 karyotype, the X-linked gene expression would differ in these two genetic scenarios. As there is only one X chromosome in the 45,XO cells, there is no chance to perform X inactivation. So the 45,XO cells would have the same expression of X-linked genes as a normal male (46,XY). And there would be similar risks as male cells if the single X chromosome had a flawed gene, such as the situation in X-linked chronic granulomatous disease in which male offspring have a faulty X-linked phagocytic nicotinamide adenine dinucleotide phosphate (NADPH) oxidase gene [22]. Without a functional NADPH oxidase gene, the males cannot clear bacterial infections and succumb at an early age.

On the other hand, the 46,XX del Xq22 cells have two possibilities depending on whether the complete X or the partial X is chosen as the inactive X (Xi). Both of the X chromosomes should have intact X inactivation centers so they can co-localize and randomly choose which X chromosome will be inactivated by persistent XIST RNA expression. If the partial X is inactivated, the normal X will become the active X and should provide proper X-linked gene expression [20]. However, there may be difficulties in maintaining partial X in an inactive state. Besides the XIST RNA, other factors help maintain XCI, such as cohesin and CCCTC-binding factor (CTCF) that are involved in establishing topologically associated domains (TADs) [20]. These may be missing due to the deletion of Xq22 to the Xq terminus. Therefore, XCI in partial X may be less stable. Failure to properly inactivate the partial X or reactivation of the partial X could lead to overexpression of genes on the partial X, such as spermidine/spermine N1 acetyltransferase (SAT1) at Xp22, an enzyme that recycles polyamines. SAT1 is located on the short arm of the X chromosome (XP) and is normally expressed only from the active X [23]. SAT1 can undergo superinduction during cellular stress. With two active copies of SAT1 due to X reactivation, there could be extensive recycling of polyamines, which can result in increased putrescine, a precursor for new polyamine synthesis. SAT1 overexpression and an increase in putrescine have been reported in synovial fibroblasts in rheumatoid arthritis [24]. Putrescine can bind an allosteric site on S-adenosylmethionine decarboxylase (AMD1) and cause increased conversion of S-adenosylmethionine (SAM) to decarboxylated SAM, needed for polyamine synthesis. This reduces the levels of SAM needed for DNA and protein methylation. As a result, there could be a disruption of epigenetic control with failure in DNA methylation needed to suppress genes.

Instability in maintaining XCI can have other consequences, even leading to mutual disruption of the inactive X chromosome and the nucleolus during cellular stress, as proposed for SLE [25]. The Xi (aka “nucleolar satellite”) is often localized with the nucleolus, even being incorporated into the nucleolar heterochromatic shell. Disruption of the Xi during a nucleolar stress response can open an abundance of Alu elements in the pseudoautosomal region 1 (PAR1) of the Xi. Then a dramatic increase of (RNA) polymerase III Alu transcripts generated from PAR1 and the rest of the genome can interfere with RNA polymerase II transcripts with intronic Alu elements that maintain the nucleolar structure leading to fragmentation of the nucleolus. In the situation where the complete X chromosome is inactivated, expression of some key genes, such as MeCP2 that are missing on the active partial X, could be fatal for that cell unless MeCP2 on the inactivated complete X chromosome can escape inactivation and provide sufficient MeCP2.

In the case of GD, the main autoantigen target is the thyrotropin receptor (aka thyroid-stimulating hormone receptor; TSHR), a G-coupled protein receptor [26]. TSHR is located on chromosome 14. We should note that chromosome 14 contains nucleolar organizing regions (NORs) that localize chromosome 14 and the TSHR gene to the nucleolus, thereby setting up a scenario in which an exceptional nucleolar stress response could potentially disrupt epigenetic control of nearby genes, including the TSHR gene leading to altered expression. This is similar to the proposed disruption of Xi described above. Another possibility for TSHR emerging as an autoantigen is a disruption of the alternate splicing that occurs with TSHR [27]. Such disruption could yield previously suppressed isoforms of TSHR that provoke an autoimmune response.

As far as other genes showing an association with GD, the CTLA-4 gene located on chromosome 2 at 2q33 has shown a more consistent association with GD susceptibility, although not as an autoantigen [28]. CTLA-4 is involved in the regulation of T cell expansion. Mutations in CTLA-4 could lead to failure in suppressing T cell expansion and subsequent interleukin expression by the T cells. Also, some human lymphocytic antigen (HLA) subtypes, especially HLA-DR3, have shown an association with GD [29].

## Conclusions

In conclusion, a stepwise increase in autoimmune diseases occurs with a progressive increase in the dosage of X-linked genes (as in 47XXX) or with an apparent progressive reduction in the dosage of X-linked genes (as in TS with 45XO) compared to normal women (with 46XX). The development of autoimmune diseases may depend on the absence of the X inactivation process or disruption of X inactivation at some point due to genetic (somatic or germ-line) or epigenetic disruption of genes on the X chromosome. Because of the close association between TS and various AITDs, clinicians should screen and evaluate AITD among children with TS, so that adequate treatment can be initiated at the early stage of the disease.
